# Responses of Oat Grains to *Fusarium poae* and *F. langsethiae* Infections and Mycotoxin Contaminations

**DOI:** 10.3390/toxins10010047

**Published:** 2018-01-20

**Authors:** Charlotte Martin, Torsten Schöneberg, Susanne Vogelgsang, Carla Susana Mendes Ferreira, Romina Morisoli, Mario Bertossa, Thomas D. Bucheli, Brigitte Mauch-Mani, Fabio Mascher

**Affiliations:** 1 Plant Breeding and Genetic Resources, Agroscope, 1260 Nyon, Switzerland; charlotte.martin@agroscope.admin.ch (C.M.); carlasusana.mendesferreira@unil.ch (C.S.M.F.); 2 Ecology of Noxious and Beneficial Organisms, Agroscope, 8046 Zürich, Switzerland; torsten.schoeneberg@agroscope.admin.ch (T.S.); susanne.vogelgsang@agroscope.admin.ch (S.V.);; 3 Plant Protection South of the Alps, Agroscope, 6593 Cadenazzo, Switzerland; romina.morisoli@agroscope.admin.ch (R.M.); mario.bertossa@agroscope.admin.ch (M.B.); 4 Environmental Analytics, Agroscope, 8046 Zürich, Switzerland; thomas.bucheli@agroscope.admin.ch; 5 Faculty of Science, University of Neuchâtel, 2000 Neuchâtel, Switzerland; brigitte.mauch@unine.ch

**Keywords:** Fusarium head blight, nivalenol, T-2/HT-2, resistance, grain properties, β-glucan, We successfully inoculated *Fusarium langsethiae* in field tests on oats, resulting in T-2/HT-2 contaminations. Compared to NIV, after artificial infections with *F. poae*, T-2/HT-2 contaminations required even higher humidity conditions. In both cases, the infection altered the β-glucan content of the grains without impacting their weight.

## Abstract

Recent increases of Fusarium head blight (FHB) disease caused by infections with *F. poae* (FP) and *F. langsethiae* (FL) have been observed in oats. These pathogens are producers of nivalenol (NIV) and T-2/HT-2 toxin (T-2/HT-2), respectively, which are now considered major issues for cereal food and feed safety. To date, the impact of FP and FL on oat grains has not yet been identified, and little is known about oat resistance elements against these pathogens. In the present study, the impact of FL and FP on oat grains was assessed under different environmental conditions in field experiments with artificial inoculations. The severity of FP and FL infection on grains were compared across three field sites, and the resistance against NIV and T-2/HT2 accumulation was assessed for seven oat genotypes. Grain weight, β-glucan content, and protein content were compared between infected and non-infected grains. Analyses of grain infection showed that FL was able to cause infection on the grain only in the field site with the highest relative humidity, whereas FP infected grains in all field sites. The FP infection of grains resulted in NIV contamination (between 30–500 μg/kg). The concentration of NIV in grains was not conditioned by environmental conditions. FL provoked an average contamination of grains with T-2/HT-2 (between 15–132 μg/kg). None of the genotypes was able to fully avoid toxin accumulation. The general resistance of oat grains against toxin accumulation was weak, and resistance against NIV accumulation was strongly impacted by the interaction between the genotype and the environment. Only the genotype with hull-less grains showed partial resistance to both NIV and T-2/HT-2 contamination. FP and FL infections could change the β-glucan content in grains, depending on the genotypes and environmental conditions. FP and FL did not have a significant impact on the thousand kernel weight (TKW) and protein content. Hence, resistance against toxin accumulation remains the only indicator of FHB resistance in oat. Our results highlight the need for new oat genotypes with enhanced resistance against both NIV and T-2/HT-2 to ensure food and feed safety.

## 1. Introduction

Fusarium head blight (FHB) is recognized as a major threat for oat production [1,2,3]. The disease is caused by different species belonging to the genus *Fusarium*, and leads to the accumulation of mycotoxins in grains that are toxic for humans and animals [4,5]. In oats from Scandinavia, the most dominant FHB-causing species is *F. graminearum*, producing deoxynivalenol (DON) [6,7,8]. However, in other European areas, a considerable increase in *F. poae* (FP) and *F. langsethiae* (FL) infections has been observed in oats [9,10,11,12,13]. These pathogens produce highly noxious toxins, putting food and feed safety in jeopardy. FP is the main producer of nivalenol (NIV), a vomitoxin that is more than 10 times more toxic than DON [14,15]. FL has been associated with the recent increases of oat grain contaminations with hazardous T-2 and HT-2 toxins [11,13]. In fact, T-2/HT-2 contaminations caused an outbreak of alimentary toxic aleukia that was responsible for the death of a thousand people in Russia in the 1940s [16]. Guidance levels have been defined to limit the amount of T-2/HT-2 entering the food chain (European Commission 2013/165/EC [17]); however, limits for NIV have not yet been defined. FP and FL are emerging pathogens, and in contrast to *F. graminearum*, very little is known about the life cycle, the epidemiology, and environmental requirements for infection and toxin production. Without such information, it remains difficult to forecast FL and FP infection, and thus avoid toxin contaminations [13].

Oat has long been regarded as more resistant to FHB than wheat or barley. This may be attributed to the lack of visual symptoms on infected panicles, and the presence of long pedicels between spikelets that usually prevent the spread of fungal mycelia throughout the panicle [6,18]. Yet, oat grains can accumulate considerable mycotoxin amounts. The use of resistant oat varieties would be the most sustainable way to overcome such contaminations [19,20]. However, the selection of FHB resistant oat varieties has been strongly delayed due to a lack of knowledge about FP and FL infection processes and their impact on oat tissues. Nevertheless, it has been demonstrated that oats, much like other small grain cereals, are susceptible to primary infection during anthesis [21,22]. Fungal hyphae enter via the tips of the glumes, quickly colonize the anthers, and then reach the developing kernels, which are frequently aborted [23,24]. Besides toxin contamination, the consequences of *Fusarium* infections on oats are not yet clearly identified. Some epidemiological studies relate a significant reduction of grain weight and germination ability [24,25], whereas others report minimal consequences on crop yields and grain properties [6,19]. Moreover, the vast majority of studies aiming to describe FHB symptoms only focus on plants infected with *F. gramineraum* or *F. culmorum* [5,6,25]. As of yet, it is not known how oat grains react to the infection with FP and FL.

As in other small grain cereals, oat resistance against FHB has a complex, quantitative character that is based on the cumulative effect of several genes [26,27]. Up to now, all of the resistance studies in oats aimed to limit the toxin contamination in grains, which is defined as type V resistance in wheat [28]. It has been observed that naked grains accumulate lower amounts of DON than hulled grains [3,29]. Recently, several minor quantitative trait loci (QTLs) associated with resistance to DON accumulation have been identified in oats [26,27]. It is not clear if resistance against DON contamination also provides resistance against NIV and T-2/HT-2 toxins. Besides resistance type V, other resistance types have not yet been described in oat. The resistance type II, which is described in wheat as the resistance against disease propagation in head tissue [30,31], is inherent to the panicle shape [6]. Due to the limited knowledge of oat grain responses to FHB, so far it has not been possible to examine the resistance against kernel infection (type III) and the tolerance of grain in terms of yield (type IV).

The aim of this study is to investigate the responses of oat grains to infection by FP and FL. Seven oat genotypes were artificially inoculated at three different field sites. Each experiment was composed of plots inoculated with either FP or FL, as well as non-inoculated plots. The success of the infections was determined by the observation of grains infected by the pathogens and measurements of fungal DNA in three varieties of grains. The contents of NIV and T-2/HT-2 toxins were measured in all of the grains from the inoculated plots. The impacts of FP and FL on grain properties were assessed by comparison of the grain weight, β-glucan contents, and protein contents between inoculated and non-inoculated grains.

## 2. Results

### 2.1. Outcome of the FP and FL Inoculations

The success of artificial inoculation on panicles for causing an infection on grains was assessed by recovering FP and FL colonies from surface sterilized grains. The outcome of inoculation from the three field sites was assessed on three selected oat genotypes (Canyon, Husky, and Triton). Canyon, Husky, and Triton are the most differing varieties from an agronomical point of view (precocity, yield, and genetic background). The percentage of *Fusarium*-infected grains and the amounts of fungal DNA were determined in grains from FP and FL inoculated plots using seed health tests (SHT) [32] and quantitative PCR [13], respectively.

**Figure 1:**
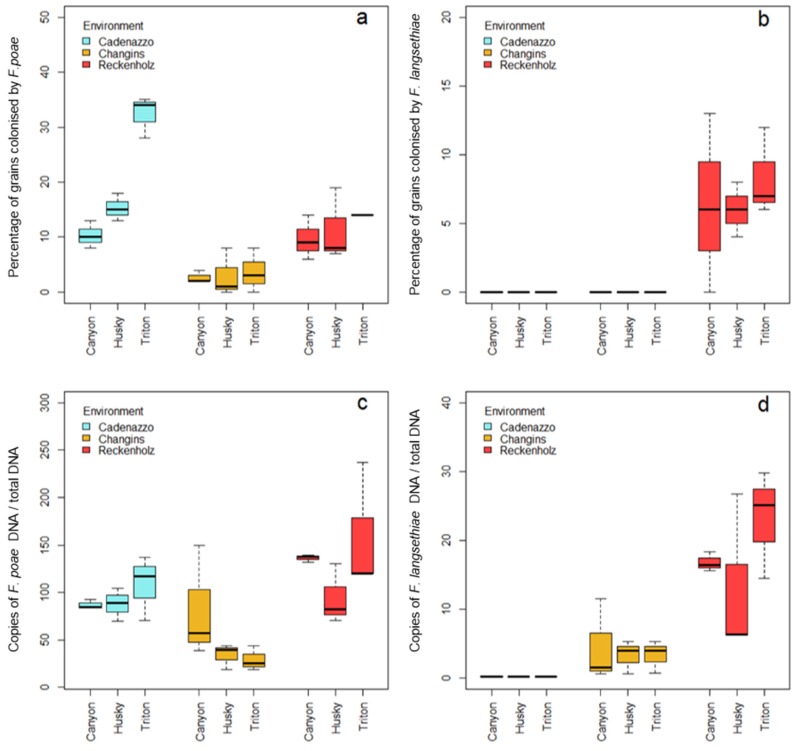
Percentage of grains by colonized *F. poae* (**a**) and *F. langsethiae* (**b**), and fungal DNA of *F. poae* (**c**) and *F. langsethiae* (**d**) for three oat genotypes from three environments (field sites) with three replicates. Cadenazzo: canton Ticino, Changins: canton Vaud, Reckenholz: canton Zürich.

After inoculation with FP, the fungus was present on grains in all of the field sites, as verified with the seed health test and through the quantification of FP DNA (Figure 1a,c). The inoculation with FL was successful only at the Reckenholz site (Figure 1b,d). FL was neither re-isolated with SHT, nor was FL DNA recovered on grain samples from Cadenazzo or Changins.

On non-inoculated control samples, the SHT revealed that less than 5% of the grains were infected by *F. graminearum* in Cadenazzo and Changins, and by the non-toxigenic *Microdochium nivale/M. majus* in Reckenholz. Similarly, in the inoculated plots, less than 5% of the grains showed the presence of *F. graminearum* and *Microdochium nivale/M. majus*.

Based on these results, grains from the three sites were used for analyses of oat grain response to FP. Grains from Reckenholz were used for subsequent assessments of FL impact on oat grains.

### 2.2. Incidence and Severity of Grain Infection with FP and FL

The percentage of *Fusarium* colonized grains and the amounts of fungal DNA were used to determine the incidence and the severity of grain infection caused by FP and FL, respectively. The incidence and severity were compared between environments and between genotypes.

The proportion of grains colonized by FP ranged from 2% to 35% (Figure 1a). Over all of the genotypes, the highest proportion of FP-colonized grains was observed in grains from Cadenazzo (20%), whereas FP was found on 12% of the grains from Reckenholz, and 3% of the grains from Changins. Yet, the FP DNA abundance in grains showed higher quantities in grains from Reckenholz than in grains from Changins and Cadenazzo (*p* < 0.05) (Figure 1c). The SHT revealed a higher FP incidence (*p* < 0.05) in Cadenazzo for grains from Triton (33%) compared with grains from Canyon and Husky grains (11% and 16%, respectively), but no differences in FP DNA quantities were detected between the three genotypes. No significant difference in the severity of grain infection was observed between the genotypes in Changins and Reckenholz. Over all of the environments, the percentage of FP-infected grains and the quantity of FP DNA in grains were significantly correlated (Pearson correlation coefficient: 0.44, *p* < 0.05).

With respect to FL inoculations, the SHT and the DNA quantifications revealed no infection in grains from Cadenazzo and Changins. Even in Reckenholz, FL caused less severe grain infections than FP. Indeed, on average, only 9% of grains were infected by FL after artificial inoculation (Figure 1b), and quantities of fungal DNA were 10 times lower than those detected in FP inoculated grains. No significant differences were observed between genotypes considering the percentage of infected grains, but significantly higher quantities (*p* < 0.05) of FL DNA were measured in Triton compared with grains from Canyon and Husky (Figure 1d). Nevertheless, the proportion of FL colonized grains, and the quantity of DNA in grains, were significantly correlated (Pearson correlation coefficient: 0.71, *p* < 0.001).

The colonization of grains and the outcome of the infection was dependent on the environmental conditions. Table 1 shows the meteorological data at the three experimental sites between flowering and harvest. The higher colonization of grains with FL in Reckenholz (Figure 1b) corresponded to the higher precipitations, higher relative humidity, lower evapotranspiration, and optimal temperatures for the infection at anthesis than at the other sites. In Cadenazzo, the highest colonization by FP on all of the varieties, in particular on Triton (Figure 1a), goes along with elevated precipitations post-anthesis. Colonization of FP was higher in Reckenholz than in Changins, which corresponds to the differences in precipitation and relative humidity. The evapotranspiration, which is an important indicator for the plant water supply, was similar in Reckenholz and Cadenazzo, but slightly higher in Changins. The experimental site in Changins was therefore drier and less conducive to infection by the FP.

### 2.3. Incidence and Severity of Grain Infection with FP and FL

Mycotoxins contents were measured in grains of all seven genotypes. NIV content was measured in grains from FP-inoculated plots and T-2/HT-2 was measured in grains from FL-inoculated plots. No mycotoxins were detected in grains from non-inoculated plots.

#### 2.3.1. NIV Contamination Caused by FP

**Figure 2:**
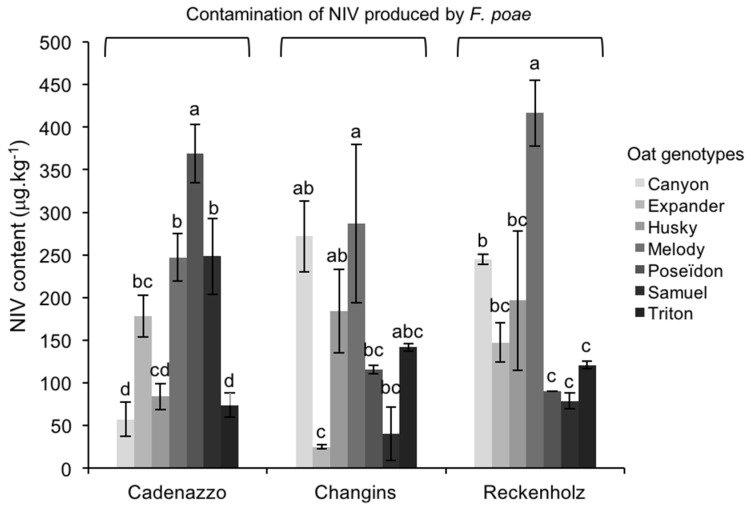
Average ± standard deviation of nivalenol (NIV) content measured by LC-MS/MS in seven oat genotypes from three environments (field sites) after artificial inoculation with *F. poae*. All of the trials were done with three replicates. Different letters indicate significant differences within one environment, according to Tukey HSD (α = 0.05).

NIV was detected in all of the grain samples infected with FP, and ranged between 30–420 μg/kg (Figure 2), with an average of 172 μg/kg. NIV accumulation was not influenced by the environment (field site); however, significant differences (*p* < 0.05) were observed in NIV between oat genotypes (Table 2). Over all the environments, the grains of Melody accumulated significantly higher (*p* < 0.05) NIV amounts (281 μg/kg) than the grains from Husky (143 μg/kg), Samuel (139 μg/kg), Expander (125 μg/kg), and Triton (117 μg/kg) (Figure 2). NIV accumulation in grains from Poseïdon and Canyon was intermediate (respectively 218 μg/kg and 170 μg/kg), and not significantly different from the other genotypes (*p* > 0.05). The accumulation of NIV in oat genotypes was subject to interactions between the variety (genotype) with the environment (GxE interactions). Indeed, in Changins and Reckenholz, Melody grains accumulated the highest NIV quantities, whereas in Cadenazzo, the highest content was measured in grains from Poseïdon (*p* < 0.05). In Cadenazzo, grains from Canyon were among the less affected by NIV contamination, yet accumulated considerable NIV amounts in Changins. These interactions between genotypes and environments (GxE interactions) explained 46% of the NIV accumulation variability in the present experiment (Table 2). No significant correlation was observed between fungal DNA and NIV contents in FP-infected grains.

#### 2.3.2. T-2/HT-2 Contamination Caused by FL

**Figure 3:**
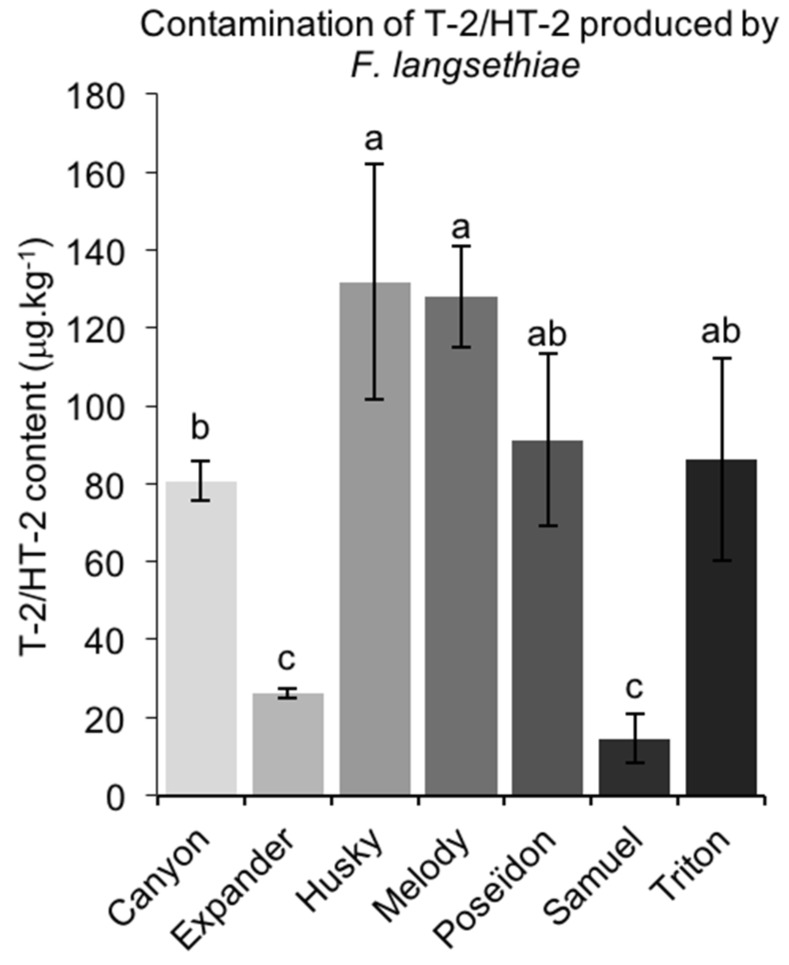
Average ± standard deviation of T-2/HT-2 toxin content measured in seven oat genotypes at Reckenholz after artificial inoculation with *F. poae*. All of the trials were done with three replicates. Different letters indicate significant differences within one environment, according to Tukey HSD (α = 0.05).

In Reckenholz, all of the grains from FL-inoculated plots contained T-2/HT-2 toxins. The highest T-2/HT-2 contents were measured in grains from Husky (132 μg/kg), and the lowest were measured in grains from Expander and Samuel (26 μg/kg and 15 μg/kg) (Figure 3). No significant correlation was observed between fungal DNA and both NIV and T-2/HT-2 contents in FL-infected grains.

### 2.4. Impact of the Infection on Grain Properties

The impacts of FP and FL infections on grain properties were assessed by analyses of grain weight, β-glucan content, and protein content. These properties were compared between grains from inoculated and non-inoculated plots. In our data set, thousand kernel weight (TKW) varied between 23.6–38.5 g, β-glucan content in grains ranged between 2.8–4.8% in dry weight, and protein content ranged between 11.7–12.9%.

#### 2.4.1. Effect of FP Infection on Grain Properties

**Figure 4:**
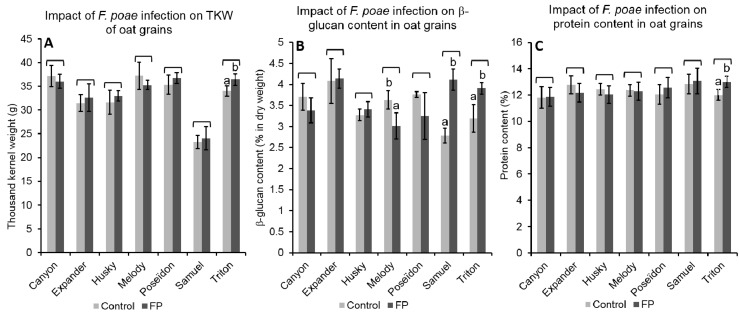
Average and standard deviations of (**A**) the thousand kernel weight (TKW), (**B**) β-glucan contents, and (**C**) protein contents in grains from plots without (Control) or with *F. poae* (FP) inoculation, for seven oat genotypes and over all three environments (field sites). All of the trials were done with three replicates. Different letters on the top of the columns indicated significant differences between infected and non-infected grains of a given genotype, according to Tukey HSD (α = 0.05).

FP infection resulted in minor changes of TKW. Only a slight increase in the TKW of Triton was observed over all of the environments (from, on average, 34.0 g for non-inoculated grains to 36.4 g for inoculated grains) (Figure 4a). Within each environment, FP inoculation had a different impact on the TKW of oat genotypes. In Changins, infection led to significant TKW increase (*p* < 0.05) in grains from Expander and Melody (31.2 g to 36.3 g and 32.6 g to 35.7 g, respectively). However, in Cadenazzo and Reckenholz, FP inoculation reduced the TKW of grains from Melody grains (43.4 g to 34.9 g), and of grains from Canyon (40.0 g to 35.5 g), respectively. Over all of the environments, FP inoculations reduced β-glucan content in grains from Melody (−17.0%), while increasing contents in grains from Samuel and Triton (47.7% and 22.0% increases, respectively) (Figure 4b). Moreover, FP inoculations led to a general increase of β-glucan content in grains from Changins over all of the genotypes (on average from 3.2% to 3.8% of β-glucan). In addition, FP inoculations resulted in a weak but significant increase of the protein content in grains from Triton over all of the environments (from 12.0% in grains from non-inoculated plots to 13.0% in grains with FP inoculation) (Figure 4c). Within each environment, significant differences in protein content were observed between infected and non-infected grains. In Reckenholz, lower protein contents were measured in FP-infected grains over all of the genotypes (*p* < 0.05) (on average 12.2% of protein in non-infected grains and 11.9% in FP-infected grains). In Cadenazzo, FP infection caused a significant increase (*p* < 0.05) of protein content in grains from Canyon, Poseïdon, and Samuel. In Changins, FP infection resulted in a significant (*p* < 0.05) increase of protein content in grains from Triton and Samuel, whereas a reduction was observed in grains from Expander (see Table S1).

The variance analyse of TKW, β-glucan content, and protein content in oat grains considered as factors the different environmental conditions in the three field sites, the presence of FP artificial inoculations, and the different genotypes. These analyses revealed that FP inoculations did not systematically impact the TKW, β-glucan content, or protein content of oat grains. Changes of TKW in FP-inoculated grains were influenced by environmental conditions and genotypes. The interactions between the environment with the genotypes (ExG), the infection with the genotype (IxG) and the triple interactions between the environment, the infection, and the genotype (ExIxG) are shown in Table 3. The variability of TKW in our data set was mainly explained by the differences between genotypes, and can be attributed to the hull-less genotype Samuel. Changes in β-glucan contents in infected grains were mainly influenced by the genotypes (Table 3) and by the environmental conditions. These IxG and ExI interactions explained a larger proportion of β-glucan content variability than TKW or protein content variabilities. Moreover, no significant ExIxG interactions on β-glucan content were observed, indicating a stability of the impact of FP on β-glucan content across the different environments for each oat genotype.

Basically, the variability of β-glucan content was partially attributed to the differences between genotypes, as well as to significant ExG interactions. Similarly, changes in protein content in FP-infected grains were also influenced by the environmental conditions and the genotypes (ExG, IxG and ExIxG interactions). Over all of the genotypes, lower protein contents were measured in grains from Reckenholz (12.0%) than in grains from Changins and Cadenazzo (12.5% and 12.6%, respectively).

#### 2.4.2. Effect of FL Infection on Grain Properties

**Figure 5:**
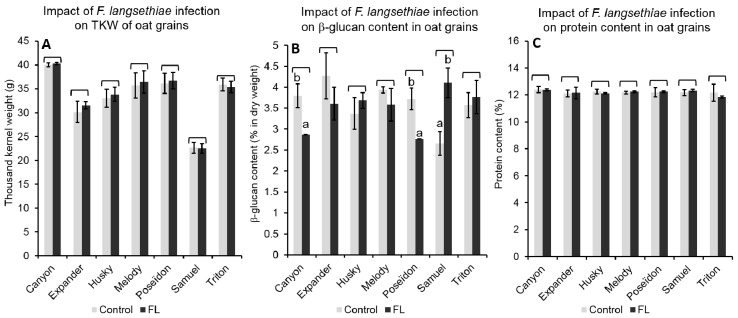
Average and standard deviations of (**A**) thousand kernel weight (TKW), (**B**) β-glucan content, and (**C**) protein content in grains from plots without (Control) or with *F. langsethiae* (FL) inoculation, for seven oat genotypes in Reckenholz (field site). All of the trials were done with three replicates. Different letters on the top of the columns indicate significant differences between infected and non-infected grains of a given genotype according to Tukey HSD (α = 0.05).

The impact of FL infections on grain properties was solely investigated in grains from Reckenholz. For all of the tested genotypes, TKW and protein content were not affected by FL inoculations (Figure 5). However, FL inoculation changed the β-glucan content in grains depending on the genotype (*p* < 0.001). FL inoculations resulted in a decrease of the β-glucan contents (*p* < 0.05) in grains of Canyon and Poseïdon (from 3.9% in grains from non-inoculated plots to 2.9% in grains from inoculated plots, and from 3.8% to 2.8%, respectively). In contrast, FL inoculation caused a significant increase of β-glucan by 63.5% in grains from Samuel (from 2.6% to 4.1%) (Figure 5).

## 3. Discussion

The presence of different types of mycotoxins in oat grains is a menace for the whole value chain. The mycotoxins found in the current study are produced by several *Fusarium* species. In particular, *F. poae* (FP) and *F. langsethiae* (FL) caused contamination of grains with highly toxic NIV and T-2/HT-2 [13,34,35], respectively. In some geographic areas, *F. graminearum* and its mycotoxins deoxynivalenol (DON) and zearalenone are also found in oat grains [6,24]. This study aims to better understand the response of oat grains in the presence of FP, FL, NIV, and T-2/HT-2 toxins. Using artificial inoculations, we intended to trigger the response of oat grains and to challenge the resistance of seven oat genotypes. Despite artificial inoculations, no symptoms were detected on the panicles in all of the experiments. This finding is in agreement with other studies on oats [2,18,36]. Severe symptoms such as blighted spikelets, the discoloration of glumes, floral abortions, and browning of rachis attributed to *Fusarium* infections are rare, and mainly observed in Northern Europe [6,37]. Divon et al. [23] observed these symptoms on oat panicle strongly infected with FL under controlled conditions. The presence of such FHB symptoms on panicles is probably due to long exposure to high humidity [24,38]. In the present study, without visible signs of FHB outbreak, the success of infections was assessed by recovering the inoculum on the grains using a seed health test (SHT) [32] and quantitative PCR for the amount of fungal DNA. Infections with FP were successful at all three field sites, whereas FL infections could only be tracked in Reckenholz. The average temperature of about 20 °C was rather similar at the three field sites, but the meteorological conditions in Reckenholz, with a high relative humidity and a long rainy period during and after anthesis, might have favored the infection by FL. Indeed, in vitro studies showed that FL requires longer periods of high humidity, compared with FP and other *Fusarium* species [39].

Once the grains are infected, both FP and FL produce mycotoxins. The infection of FP caused an accumulation of NIV in similar contents across all of the field sites. In more contrasting environments across the United Kingdom and Canada, Edwards et al. [10] and Tekauz et al. [18], respectively, noticed the strong influence of environmental conditions on toxin accumulation in grains. In the present study, it was not possible to investigate the impact of environmental conditions on T-2/HT-2 accumulation. T-2/HT-2 were detected in considerable amounts in all of the FL-infected grains from Reckenholz, with a maximum value of 180 μg/kg. Yet, the results of the SHT and the quantification of FL DNA revealed a low degree of kernel colonization by FL, whereas FP caused more severe grain infections. This result suggests a high toxigenicity of FL and indicates that FL infections on oat crops, although less dominant than FP, nevertheless constitute a real threat to the safety of oat products. In plants, T2/HT2 are transformed in a glycosylated form [40] that may not be detectable by the ELISA kit (manufacturer, personal communication).

Besides the accumulation of toxins, FP and FL altered the β-glucan content in grains. Depending on the genotypes and environmental conditions, the β-glucan contents increased or decreased subsequent to FP and FL infections. In particular, the β-glucan content in grains of the naked variety Samuel substantially increased following infections by both FP and FL (+48% and +64%, respectively), whereas the changes in β-glucan content caused by FHB were limited for the other genotypes (approximately +20%). Hence, with the current data set, no clear tendency in β-glucan content variation could be identified. In a very similar study on barley grains, a 10% reduction of β-glucan content was observed in six barley varieties after inoculation with *F. graminearum* [41]. On one hand, the reduction of β-glucan content might be attributed to the β-glucanase activity observed from *Fusarium* pathogens [42]. On the other hand, since β-glucan are mainly concentrated in the outer layers of oat grains [43], such variations in β-glucan content might be attributed to changes in grain morphology caused by the infection. With respect to the TKW of grains, FP and FL infections showed only a weak impact in the present study. Over all three environments, only a weak increase of TKW of grains from Triton was measured when infected with FP. This indicates that both FP and FL had very little to no impact on grain filling, suggesting a minimal impact of FHB on oat yield. However, these observations are likely conditioned by the prevailing environmental conditions in Switzerland. In Brazil, with temperatures above 20 °C and relative humidity frequently exceeding 90%, Martinelli et al. [44] observed a significant reduction of oat yield, up to 25%, due to FHB caused by *F. graminearum*. Furthermore, Mielniczuk et al. [25] detected TKW reductions of 32% in oats after field inoculations with *F. crookwellense* in Poland. We also assume that a reduction of grain filling and yield is specific to the particular *Fusarium* species causing FHB. In the present study, we did not observe a significant impact of FP and FL on the protein content in oat grains. Finally, our grain analyses showed that even if FP and FL have an impact on some of the grain properties, these alterations are not reliable and useful symptoms of FHB in breeding for resistant varieties.

For the understanding of resistance against FHB in oats, we compared the types of resistance with those for wheat, where type I and type II comprise the resistance against the primary infection and the spread of the infection throughout the spike, respectively [30,31]. In the present experiments with oats, we could not distinguish these types of resistance. Types III to VI describe the resistance of the kernel, where type III is the resistance against colonization, type IV is the tolerance against FHB in terms of yield, and type V is the resistance against the accumulation of trichothecenes [28,30]. The three cultivars Canyon, Husky, and Triton displayed a similar degree of fungal incidence, and hence, a type III resistance was not detected. Nevertheless, Gagkaeva et al. [36] noticed significant differences in *F. sporotrichioides* DNA in oat grains among 105 genotypes, indicating the presence of type III resistance elements that remains to be investigated. In the present study, there was no significant weight loss or changes in TKW between the varieties and therefore, no detectable type IV resistance. Among the panel of oat cultivars, none of them was able to completely avoid contaminations with NIV or T-2/HT-2 (type V resistance). Nevertheless, we observed significant differences in toxin accumulation between the genotypes. This result is consistent with Yan et al. [3], who previously suggested that, to date, oat FHB resistance can only be reflected by type V resistance. However, the authors detected only small differences in mycotoxin accumulation between the genotypes, and none displayed a high type V resistance. The general susceptibility of oat to toxin contamination was also reported in several previous studies on both modern oat varieties and oat landraces [18,29,36,45,46]. In the current study, we observed that resistance of oat genotypes to toxin contamination was strongly impacted by environmental conditions, with strong GxE interactions explaining 46% of NIV accumulation in grains infected with FP. This finding suggests a weak stability of resistance in oat genotypes, which could impede the breeding for FHB resistance. Moreover, when comparing T-2/HT-2 and NIV accumulation in FL and FP-infected grains from Reckenholz, we noticed that the variety Triton did not accumulate NIV, whereas considerable amounts of T-2/HT-2 toxins (90 μg/kg) were detected. In fact, Edwards et al. [10] found no correlation between HT-2 and DON content in oat grains. Hence, it is likely that type V resistance depends on the nature of the toxin, and the QTLs associated with low DON accumulation [26,27] will not provide enhanced resistance to other mycotoxins. In our study, only the variety Samuel was resistant to both T-2/HT-2 and NIV across all field sites. This variety produces naked grains, suggesting a role of hulls in mycotoxin accumulation [29,36,45]. Interestingly, in grains from Samuel, we also measured high increases of β-glucan content. We suggest that this reaction of the grain also contributes to the low accumulation of toxins.

Overall, the results presented here show that oats at the panicle level are constitutively resistant to FHB. However, the pathogen colonizes the glumes and grains depositing mycotoxins in the developing grain. Only a weak resistance against mycotoxin accumulation was observed, which was probably due to the absence of glumes (naked grains) in the variety Samuel. Improving resistance against mycotoxin accumulation may be achieved by the use of resistant wild oats (i.e., *Avena sterilis*) and other germplasms as new source of resistance for breeding [6,46].

## 4. Materials and Methods

### 4.1. Plant Material

Seven oat varieties were used for this study. The varieties differ in their morphological properties, favored environmental conditions for cultivation, and resistance to DON accumulation (Stefan Beuch, personal communication). Plant height, panicle characteristics, and grain characteristics are presented in Table 4 [47]. Descriptions of these characteristics were carried out in the field at Changins in 2015. Ten plants of each variety from non-inoculated plots were randomly chosen, and the averages of plant height and number of seeds in a panicle were calculated.

### 4.2. Fungal Material

Artificial inoculations were carried out with three strains of FL and three strains of FP, all isolated from infected oat grains (Table 5).

Strains were retrieved from long-term storage in a 1:1 mix of water with glycerol at −80 °C. For the mass production of each isolate, strains were cultured on potato dextrose agar (PDA, BD Difco, Le Pont de Claix, France) for one week at 18 °C with 12 h/12 h UV light/darkness. Subsequently, two discs (5-mm diameter) from the outer margin of a well-grown colony were transferred to 200 mL of liquid V8-medium in a 1L capacity Erlenmeyer flask. A V8 medium consisted of a 1:5 mix of V8 juice (Campbell Soup Company, Camden, NJ, USA) and distilled water with 2 g sodium carbonate per liter. Cultures were incubated on a shaker at 200 rpm for 7 days at 24 °C in the dark. Cultures were then filtered through sterile cheesecloth to remove all mycelia. Finally, the culture medium was removed by centrifugation at 4500 rpm for 10 min, and the pellet was re-suspended in sterile distilled water. These preparations were either used immediately or stored at −20 °C.

### 4.3. Field Experiments and Artificial Inoculations

Field experiments were conducted in 2015 at three locations in Switzerland: Changins (Canton Vaud, southwest [46°24′36′′/6°14′06′′]), Reckenholz (Canton Zürich, northeast [47°16′30′′/8°26′45′′]), Cadenazzo (Canton Ticino, south of the Alps [46°09′00′′/8°57′00′′]). In Changins and Reckenholz, oat grains were sown in March 2015; in Cadenazzo, oat grains were sown in November 2014. At all of the field sites, planting and management was conducted with the same experimental design and protocol. The varieties were sown with a Seedmatic seeding machine (Hege Maschinen, Eging am See, Germany) in 1 m^2^ microplots with five rows and 15 cm space between rows. Artificial inoculations took place when 50% of the plants within each microplot were at mid-anthesis (BBCH65). Inoculum was prepared immediately before the inoculation by mixing an equal proportion of the liquid culture of each of the three strains of FP or FL, and adding 0.0125% of Tween^®^20 (Sigma-Aldrich Chemie GmbH, Darmstadt, Germany). A concentration of conidia and volume of prepared suspension were adjusted to 7.5 × 10^6^ conidia per microplot. Suspensions were applied with a hand-held sprayer (Spray-Matic 1.25P, Birchmeier, Stetten, Switzerland) at dawn. If required and according to weather conditions during inoculations, the plots were irrigated to maintain humidity on the panicles for at least 24 h, with a maximum of 600 L of water per hectare. A high pressure/low volume overhead spray irrigation system was available in Changins, while in Cadenazzo, an equivalent of 300 L/ha of water was sprayed manually with a backpack sprayer. Additional irrigation was not required at the Reckenholz site. The additional irrigation enhanced the infection pressure. The humidity regime for the plants increased by 30 mL/m^2^, which was an insignificant change in the humidity regime for the plants. Three artificial inoculations were carried out at three-day intervals. 

The meteorological conditions between anthesis and harvest were measured with the meteorological stations of Meteoswiss [33] based at each experimental site. The meteorological stations were at a distance of 500 m (Changins and Reckenholz) and 150 m (Cadenazzo). 

### 4.4. Harvest and Sample Collection

All of the oat plots were harvested at full maturity (BBCH 89) during the first week of August 2015 at the three field sites. A combine harvester (HEGE 140, Eging am See, Germany) was used, with a reduced airflow to recover a maximum of kernels. Grains were dried to a maximum of 14% moisture, and cleaned using a vertical airflow (Baumann Saatzuchtbedarf, Waldenburg, Germany) to remove dust and other debris. At this stage, clean grains with husks were obtained. Two hundred gram grain samples were extracted after three times homogenization with a rifle divider (Schieritz & Hauenstein AG, Laufen, Switzerland). Fifty grams were extracted from these subsamples and milled separately, with a sample mill using a 0.25 mm screen (1093 Cyclotec Sample Mill, FOSS, Höganäs, Sweden) to obtain whole meal flour samples. All of the grain and flour samples were stored at −20 °C until further analyses.

### 4.5. Analyses of Grain Infection

The presence of FP and FL on oat grains confirmed the success of the artificial inoculations. The infection of grains was assessed by the incidence of grains infected by FP and FL (%), and by the quantification of fungal DNA in grains.

#### 4.5.1. Proportion of Grains Infected by *F. poae* and *F. langsethiae*

The proportion of grains colonized with *Fusarium* was determined using the seed health tests method described in Vogelgsang et al. [32] and used in Schöneberg et al. [13] and Martin et al. [41]. Essentially, the seed health test consists in the culture of surface sterilized grains on the *Fusarium* selective media containing pentachloronitrobenzene [13]. For three to 10 days, the cultures were observed, and growing Fusaria were scored and taxonomically identified. Samples from the varieties Canyon, Husky, and Triton from both inoculated and non-inoculated plots were analyzed to determine the infection of grains not only from FP and FL, but also from other *Fusarium* species. The different *Fusarium* species were identified according to the *Fusarium* laboratory manual by Leslie and Summerell [49].

#### 4.5.2. Quantification of Fungal DNA

The severity of FP and FL infection was measured by quantitative PCR (qPCR) of fungal DNA in the grains. Protocols for DNA extraction, total DNA quantification, and quantitative PCR reactions are described in Schöneberg et al. [13]. In every performed assay, all standards as well as the negative control (double distilled water) were run as triplicates.

Briefly, for FP qPCR, the primer pairs ACL1-F160 and ACL1-R330 and TaqMan-Probe (ACL1_poae1_probe) (Qiagen AG, Hombrechtikon, Switzerland) were used. For FL qPCR, the protocol specifications and thermocycling parameters were employed, as described in Edwards et al. [50], and adapted to the available reaction mixes and laboratory devices as detailed in Schöneberg et al. [13]. The amplification mix consisted of the primer pairs FlangF3 and LanspoR1 [51] and IQ SYBR^®^ Green Supermix (Bio-Rad, Cressier, Switzerland).

### 4.6. Mycotoxin Analysis

NIV contents were examined in all of the samples from plots inoculated with FP, and three non-inoculated plots. The identification and quantification of NIV was done using LC-MS/MS. Sample preparation and LC-MS/MS measurements followed the protocols detailed in Schöneberg et al. [13].

Contents in T-2/HT-2 toxins in grains were measured in all of the samples from plots inoculated with FL, and three non-inoculated plots. Contents were determined in flour using an enzyme immunoassay kit for the quantitative screening of toxins (Ridascreen^®^ T-2/HT-2 Toxin, R-biopharm AG, Darmstadt, Germany), according to the manufacturer’s instructions. The protocol specific for the extraction of T-2/HT-2 in oat flour was followed. The detection limit was 30 μg/kg, according to the manufacturers’ manual.

All of the mycotoxin concentrations in grains were expressed as μg/kg of dry weight. 

### 4.7. Analyses of Grain Properties

Grain weights were measured for all of the grain samples using the thousand kernel weight (TKW) indicator. TKW were measured with the MARVIN optical grain counter (Digital Seed Analyser, GTA Sensorik GmbH, Neubrandenburg, Germany) and a balance (Mettler PM2000, Mettler-Toledo, Greifensee, Switzerland).

The content of β-glucan was determined in all of the samples (inoculated and non-inoculated plots) with the mixed-linkage β-glucan kit (Megazyme International Ireland Ltd., Wicklow, Ireland). The assay procedure of mixed-linkage β-glucan in oat flour—streamlined method—(ICC Standard Method No. 166 [52]) was followed and adapted to the laboratory facilities.

The protein contents (%) were examined in all of the samples of hulled oat grains using near-infrared reflectance spectroscopy (NIRS) with a NIRFlex N-500 (Büchi Labortechnik AG, Flawil, Switzerland). The protein calibration of NIRFlex was regularly adjusted, and basis analyses were made with the Kjeldahl method, according to ICC standard method No.105/2 [53]. The coefficient of confidentially of the calibration was R^2^ = 0.61 (C. Brabant and C. Oberson, personal communication).

### 4.8. Experimental Design and Statistical Analyses

Field design, inoculation methodology, harvest preparations and sample preparations were similar across the three field sites. Each field site consisted of three replicates with FP inoculations, three with FL inoculations, and three without artificial inoculations, planted in a split-plot design. Proportion of FP and FL-infected grains and measurements of fungal DNA were carried out on grains from inoculated plots of the three field sites for three selected oat genotypes (Canyon, Husky, and Triton). Mycotoxin measurements were performed on grains from inoculated plots in the three field sites for all seven oat genotypes. TKW, β-glucan content, and protein content were measured in all of the grain samples.

Statistical analyses were carried out using the statistical software R [54]. The effect of FP and FL infection on grains were separately analyzed. Analyses of variance ANOVA were performed on the proportion of grains infected by FP or FL, fungal DNA, and mycotoxin contents using the environment (field site) as the main factor, and the genotypes as the sub-factor. Data were normally distributed. The impact of FP or FL infections on TKW, β-glucan content, and protein content were analyzed by ANOVA using the environment as the main factor, the presence of artificial inoculation as the sub-plot, and the genotypes as the sub-sub-plots. After ANOVA, a multiple comparison was performed on significant factors using Tukey’s test with β-glucan (package “agricolae”, De Mendiburu [55]. Pearson correlations were used to verify correlations between toxin contents, fungal DNA concentration, and the proportion of *Fusarium*-infected grains.

## Figures and Tables

**Table 1:** Average temperature, sum of precipitation, relative humidity, and evapotranspiration at the three field sites recorded during anthesis (15–30 June 2015), and from anthesis until harvest (15 June–10 August 2015) [33].

	Temperature (°C)	Precipitation (mm)	Relative Humidity (%)	Evapotranspiration (mm/day)
from 15 June 2015 to 30 June 2015				
Cadenazzo	20.6	32.1	66.8	3.5
Changins	19.1	21.4	58.9	4.4
Reckenholz	17.0	61.4	71.8	2.8
from 15 June 2015 to 10 August 2015				
Cadenazzo	23.4	129.7	67.8	3.1
Changins	22.1	95.4	56.3	4.8
Reckenholz	20.9	114.7	65.5	3.7

**Table 2:** Variance analysis of nivalenol (NIV) contents in oat grains infected with *F. poae* from three environments and seven varieties. *** significant at *p* < 0.001.

Source of Variation	Sum Square	Mean Square	Significance
Environment	13,410	6705	
Error (a)	2137	534	
Genotype	188,457	31,409	***
Environment x Genotype	344,011	28,668	***
Error (b)	225,612	6267	

**Table 3:** Analyses of variance of thousand kernel weight (TKW), β-glucan content, and protein content in grains between the different factors investigated in this study (environment, presence of inoculation with *F. poae*, and genotype) as well as their interactions. Significance levels: *** at *p* < 0.001, ** at *p* < 0.01, * at *p* < 0.05.

	TKW			β-Glucan Content			Protein Content		
Source of Variation	Sum Square	Mean Square	Significance	Sum Square	Mean Square	Significance	Sum Square	Mean Square	Significance
Environment E	4.6	2.3		0.5	0.3		6.4	3.2	*
*Error (a)*	25.6	6.4		0.2	0.04		1.9	0.4	
Inoculation I	0.5	0.5		0.4	0.4		0.3	0.3	
ExI	97.8	48.9	***	2.3	1.4	***	3.6	1.8	*
*Error (b)*	9.7	1.6		1.5	0.25		1.5	0.2	
Genotype G	2339.3	390	***	7.6	1.3	***	12.1	2.0	***
ExG	248.4	20.7	***	6.9	0.6	**	20.0	1.7	***
IxG	45.5	7.6	**	13.2	2.2	***	7.5	1.3	***
ExIx G	95.7	8.0	***	2.7	0.2		10.3	0.9	***
*Error (c)*	166.3	2.3		16.9	0.2		9.3	0.1	

**Table 4:** Description of the seven oat genotypes investigated in the current study. All of the quantitative data are the average of three replicates.

Variety	Breeder	Plant Height (cm)	Panicle Shape	Panicle Erectness	Number of Seeds in a Panicle	Kernel Covering	Lemma Colour	Hairiness of Lemma	Hairiness at Basal Part of the Primary Grain
Canyon	NORDSAAT Saatzucht (DE)	110	Equilateral	Drooping	71	Covered	Yellow	Glabrous	Slightly pubescent
Expander	SZ Edelhof (DE)	105	Equilateral	Drooping	96	Covered	Yellow	Glabrous	Highly pubescent
Husky	NORDSAAT Saatzucht (DE)	110	Equilateral	Drooping	90	Covered	Yellow	Slightly pubescent	Moderately pubescent
Melody	NORDSAAT Saatzucht (DE)	110	Unilateral	Drooping	69	Covered	White	Slightly pubescent	Moderately pubescent
Poseidon	NORDSAAT Saatzucht (DE)	115	Equilateral	Drooping	85	Covered	Yellow	Slightly pubescent	Moderately pubescent
Samuel	Nufarm Deutschland GmbH (DE)	120	Equilateral	Drooping	65	Naked	/	/	Slightly pubescent
Triton	NORDSAAT Saatzucht (DE)	105	Equilateral	Semi-erected	62	Covered	White	Highly pubescent	Moderately pubescent

**Table 5:** Description of *Fusarium* strains used for the study. All of the strains were deposited at Centraal Bureau voor Schimmelcultures CBS [48].

Species	Strain ID	Origin (Swiss Canton)	Isolation Year	Toxin Produced
*F. poae*	13013	Luzern	2013	NIV
*F. poae*	13045	Thurgau	2013	NIV
*F. poae*	13059	Vaud	2013	NIV
*F. langsethiae*	13014	Jura	2013	T-2/HT-2
*F. langsethiae*	13005	Schaffhausen	2013	T-2/HT-2
*F. langsethiae*	14001	Vaud	2014	T-2/HT-2

## Supplementary Materials

The following are available online at www.mdpi.com/2072-6651/10/1/47/s1, Table S1: Average ± standard deviation of thousand kernel weight, β-glucan content and protein content for seven oat genotypes in three environments (Cadenazzo, Changins and Reckenholz), and presence of three treatments.
