# Supplementary material for: Responses of Oat Grains to Fusarium poae and F. langsethiae Infections and Mycotoxin Contaminations
Source: Toxins (Basel). 2018 Jan 20;10(1):47. doi: 10.3390/toxins10010047 (PMC5793134; doi:10.3390/toxins10010047)
Supplement: Supplementary file 1 [file toxins-10-00047-s001.pdf]

Supplementary Materials: Responses of Oat Grains to *Fusarium poae* and *F. langsethiae* Infections and Mycotoxin Contaminations

Charlotte Martin, Torsten Schöneberg, Susanne Vogelgsang, Carla Susana Mendes Ferreira, Romina Morisoli, Mario Bertossa, Thomas D. Bucheli, Brigitte Mauch-Mani and Fabio Mascher

Table S1. Average ± standard deviation of thousand kernel weight, β-glucan content and protein content for seven oat genotypes in three environments (Cadenazzo, Changins and Reckenholz), and presence of three treatments, FP: artificial inoculation with *F. poae*, FL: artificial inoculation with *F. langsethiae*, and absence of artificial inoculations (control). All trials were done with 3 replicates.

Genotype	Treatment	Thousand Kernel Weight (g)	β-glucan Content (%)	Protein Content (%)
		Cadenazzo		
Canyon	Control	36.80 ± 0.83	4.04 ± 0.53	11.37 ± 0.31
Expander	Control	32.57 ± 0.63	4.61 ± 0.37	12.67 ± 0.29
Husky	Control	32.53 ± 0.87	3.37 ± 0.39	13.03 ± 0.37
Melody	Control	43.37 ± 1.94	3.49 ± 0.50	12.47 ± 0.17
Poseidon	Control	35.43 ± 2.09	3.70 ± 0.25	11.70 ± 0.14
Samuel	Control	24.20 ±1.69	2.66 ± 0.28	13.37 ± 0.38
Triton	Control	31.70 ±0.96	3.56 ± 0.08	11.40 ± 0.22
Canyon	FP	37.37 ± 0.63	3.65 ± 0.47	12.53 ± 0.30
Expander	FP	31.83 ± 1.11	3.84 ± 0.41	12.99 ± 0.02
Husky	FP	33.43 ± 0.09	3.22 ± 0.12	12.87 ± 0.08
Melody	FP	34.90 ± 1.56	2.74 ± 0.55	12.82 ± 0.25
Poseidon	FP	36.63 ± 0.66	2.88 ± 0.39	13.10 ± 0.25
Samuel	FP	23.70 ± 0.45	3.97 ± 0.30	12.95 ± 0.04
Triton	FP	30.87 ± 1.03	3.72 ± 0.28	12.46 ± 0.35
		Changins		
Canyon	Control	34.71 ± 0.35	3.28 ± 0.07	11.67 ± 0.27
Expander	Control	31.21 ± 1.06	3.36 ± 0.23	13.56 ± 0.36
Husky	Control	29.33 ± 2.60	3.08 ± 0.16	12.02 ± 0.43
Melody	Control	32.60 ± 0.22	3.46 ± 0.21	12.46 ± 0.19
Poseidon	Control	34.53 ± 1.52	3.85 ± 0.59	12.25 ± 0.71
Samuel	Control	23.00 ± 0.79	3.03 ± 0.79	13.01 ± 0.34
Triton	Control	35.20 ± 1.07	2.44 ± 0.22	12.52 ± 0.24
Canyon	FP	35.23 ± 1.76	2.97 ± 0.32	11.30 ± 0.72
Expander	FP	36.27 ± 0.39	4.40 ± 0.17	11.79 ± 0.19
Husky	FP	33.50 ± 1.06	3.34 ± 0.47	11.34 ± 0.44
Melody	FP	35.67 ± 0.41	2.85 ± 0.22	12.53 ± 0.41
Poseidon	FP	37.77 ± 1.24	4.05 ± 0.98	12.36 ± 0.69
Samuel	FP	24.23 ± 0.60	4.47 ± 0.37	14.22 ± 0.03
Triton	FP	36.77 ± 1.21	3.97 ± 0.17	14.34 ± 0.04
		Reckenholz		
Canyon	Control	40.00 ± 0.41	3.79 ± 0.28	12.40 ± 0.24
Expander	Control	30.13 ± 2.20	4.27 ± 0.55	12.11 ± 0.24
Husky	Control	33.00 ± 1.85	3.37 ± 0.38	12.26 ± 0.17
Melody	Control	35.70 ± 2.62	3.94 ± 0.07	12.18 ± 0.09
Poseidon	Control	36.10 ± 2.12	3.72 ± 0.26	12.19 ± 0.34
Samuel	Control	22.63 ± 1.16	2.66 ± 0.28	12.18 ± 0.22
Triton	Control	35.90 ± 1.34	3.57 ± 0.30	12.15 ± 0.65

Canyon	FP	35.50 ± 0.78	3.52 ± 0.43	11.81 ± 0.29
Expander	FP	29.77 ± 1.60	4.16 ± 0.49	11.77 ± 0.16
Husky	FP	32.00 ± 0.99	3.65 ± 0.07	11.93 ± 0.29
Melody	FP	35.20 ± 0.00	3.45 ± 0.19	11.50 ± 0.40
Poseidon	FP	35.80 ± 0.00	2.83 ± 0.00	12.24 ± 0.00
Samuel	FP	22.00 ± 0.08	3.89 ± 0.23	12.04 ± 0.03
Triton	FP	35.37 ± 1.55	4.03 ± 0.54	12.10 ± 0.37
Canyon	FL	40.25 ± 0.20	2.87 ± 0.00	12.37 ± 0.09
Expander	FL	31.53 ± 0.74	3.61 ± 0.39	12.14 ± 0.43
Husky	FL	33.83 ± 1.47	3.68 ± 0.19	12.11 ± 0.05
Melody	FL	36.40 ± 2.30	3.58 ± 0.39	12.24 ± 0.05
Poseidon	FL	36.67 ± 1.70	2.76 ± 0.00	12.23 ± 0.07
Samuel	FL	22.50 ± 1.02	4.10 ± 0.36	12.32 ± 0.10
Triton	FL	35.37 ± 1.14	3.77 ± 0.39	11.84 ± 0.07
